# Glyceollins Trigger Anti-Proliferative Effects in Hormone-Dependent Aromatase-Inhibitor-Resistant Breast Cancer Cells through the Induction of Apoptosis

**DOI:** 10.3390/ijms23052887

**Published:** 2022-03-07

**Authors:** Rashidra R. Walker, Jankiben R. Patel, Akash Gupta, A. Michael Davidson, Christopher C. Williams, Florastina Payton-Stewart, Stephen M. Boué, Matthew E. Burow, Rahul Khupse, Syreeta L. Tilghman

**Affiliations:** 1 Division of Basic Sciences, College of Pharmacy and Pharmaceutical Sciences, Florida A&M University, 1415 S. Martin L. King Jr. Blvd., Tallahassee, FL 32307, USA; rashidra1.walker@famu.edu (R.R.W.); jankiben1.patel@famu.edu (J.R.P.); michael.davidson@famu.edu (A.M.D.); 2 Department of Medicine, University of Arizona, 1500 N. Campbell Ave., Tucson, AZ 85724, USA; akashg@email.arizona.edu; 3 Division of Basic Sciences, College of Pharmacy, Xavier University of Louisiana, 1 Drexel Dr., New Orleans, LA 70125, USA; cwilli35@xula.edu; 4 Division of Mathematics and Physical Sciences, College of Arts and Sciences, Xavier University of Louisiana, 1 Drexel Dr., New Orleans, LA 70125, USA; flpayton@xula.edu; 5 Southern Regional Research Center, United States Department of Agriculture, Agricultural Research Service, 1100 Robert E. Lee Blvd., New Orleans, LA 70124, USA; steve.boue@usda.gov; 6 Section of Hematology and Medical Oncology, School of Medicine, Tulane University, New Orleans, LA 70112, USA; mburow@tulane.edu; 7 College of Pharmacy, The University of Findlay, 1000 N. Main St., Findlay, OH 45840, USA; khupse@findlay.edu

**Keywords:** breast cancer, glyceollins, lapatinib, aromatase, letrozole resistance

## Abstract

Aromatase inhibitors (AIs) are standard treatment for estrogen-dependent postmenopausal breast tumors; however, resistance develops leading to tumor relapse and metastasis. We previously demonstrated that glyceollin inhibits proliferation, survival, and migration of hormone-independent letrozole-resistant breast cancer. Since many AI-resistant tumors remain hormone-dependent, identifying distinctions between estrogen-receptor-positive (ER+) and ER-negative (ER-) AI-resistant tumor response to therapy is critical. We hypothesize that treating ER+ letrozole-resistant T47D breast cancer cells (T47DaromLR) with a combination of 10 μM glyceollin and 0.5 μM lapatinib (a dual EGFR/HER2 inhibitor) will decrease cell proliferation through induction of apoptosis. The T47DaromLR cells were found to overexpress HER2 and MAPK while maintaining aromatase and ER levels compared to their letrozole-sensitive (T47Darom) counterparts. In the absence of estrogen stimulation, glyceollin ± lapatinib had no effect on the proliferation of the T47Darom cells, while glyceollin treatment caused 46% reduction in the proliferation of T47DaromLR cells, which was further diminished when combined with lapatinib. While neither agent influenced cell migration, glyceollin and lapatinib reduced S and G2/M phase cell entry and exclusively induced apoptosis by 1.29-fold in the T47DaromLR cells. Taken together, these results suggest that glyceollins and lapatinib may have potential as a novel combination therapeutic approach for hormone-dependent, letrozole-resistant tumors.

## 1. Introduction

**Figure 1:**
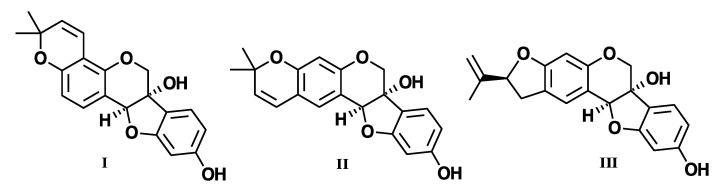
Chemical structures of Glyceollin I, Glyceollin II, and Glyceollin III.

Currently, aromatase inhibitors (AIs), such as letrozole, are first line of therapy for ER+ postmenopausal breast cancer patients. However, after five years of treatment, many patients acquire resistance [1,2,3]. It has been demonstrated that as the tumors progress from letrozole-sensitive to letrozole-refractory, they are associated with increased growth factor receptor signaling (i.e., EGFR and HER2) [4] and enhanced proliferation and motility [5]. As a result, lapatinib, an FDA-approved dual EGFR/HER2 inhibitor, was approved for use in combination with letrozole for hormone receptor (HR+) and HER2+ metastatic breast cancer patients [6]. While these therapies are effective, resistance to endocrine therapy still develops and remains a clinical obstacle. Therefore, there is an eminent need to understand additional mechanisms of resistance to develop appropriate targeted approaches. Recently, phytoestrogens have gained interest due to their antiestrogenic properties. Previously, we explored the use of glyceollin, a naturally occurring anti-estrogen from the soy plant, for the treatment of postmenopausal metastatic breast cancer that has transitioned from ER+ to ER- and demonstrated that glyceollin is capable of inhibiting the proliferation and motility in ER- letrozole-resistant breast cancer cells (LTLT-Ca) [7]. In addition, pre-clinical studies have shown that glyceollins (Figure 1) exhibit anti-proliferative effects in triple-negative breast cancer cells [8], prostate cancer cells [9], and smooth muscle cells [10]; however, the precise mechanism remains unclear. As many AI-resistant tumors are heterogeneous, we were specifically interested in understanding the subset of tumors that express an ER/PR/aromatase molecular signature. About 50% of endocrine resistance cases are associated with an ESR1 mutation; other mechanisms, increasingly uncovered, include alterations in the PI3K-AKT-mTORC1, RAS-MAPK, and CDK4/6-RB-E2F pathways and ESR1 loss, amplification, and translocation [11]. In fact, many groups have shown that during long-term estrogen deprivation, the ER is up-regulated and constitutively activated in AI-resistant breast cancer cells [12,13,14]. Here, we used a previously generated aromatase-overexpressing T47D cell that was letrozole-sensitive (T47Darom) or letrozole-resistant (T47DaromLR) to test the hypothesis that targeting ER and HER2 will trigger anti-proliferative effects selective to letrozole-resistant breast cancer cells.

## 2. Results

### 2.1. Letrozole-Resistant T47D Cells Are Codependent on Hormone and Growth Factor Signaling Cascades

**Figure 2:**
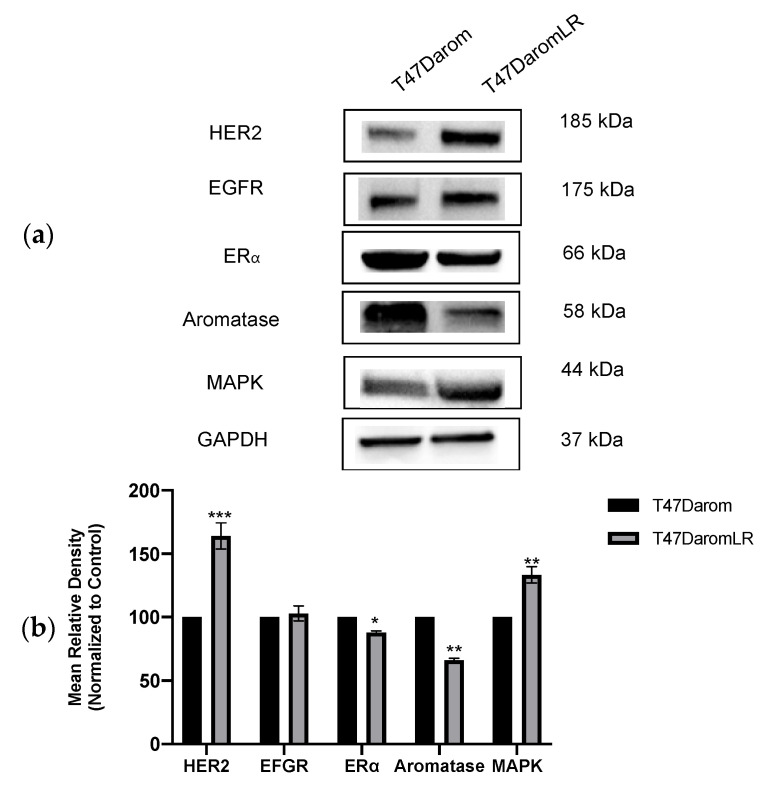
Comparative protein expression profile of letrozole-sensitive cells (T47Darom) versus letrozole-resistant cells (T47DaromLR). (**a**) The T47Darom and the T47DaromLR cells were assayed by immunoblot with antibodies directed against HER2, EGFR, ERα, aromatase, and MAPK. GAPDH was measured as an internal loading control. (**b**) Quantitative analysis representing the fold changes of the indicated proteins after normalizing to GAPDH loading control. The T47Darom cells were set to 100%. Student’s *t*-tests were performed, and treatments were compared to DMSO control. Results are expressed as the mean unit ± SD (*** *p* < 0.001, ** *p* < 0.01, * *p* < 0.05), and data are representative from one of at least three independent experiments.

Previous research demonstrated that as hormone-dependent, letrozole-sensitive breast cancer cells transition to a hormone-independent and letrozole-resistant phenotype, they utilize growth factor signaling pathways such as EGFR [5], p38/MAPK [4,15,16], and HER2 [17] as a mechanism of survival. This transition was associated with migration, EMT, and a more aggressive phenotype. However, it is unclear how cells respond if they remain hormone-dependent while acquiring resistance to letrozole. To investigate changes in protein expression between hormone-dependent letrozole-sensitive cells (T47Darom cells) and hormone-dependent letrozole-resistant cells (T47DaromLR cells), immunoblot analysis was performed. Compared to the T47Darom cells, aromatase and ERα expression was slightly reduced in the T47DaromLR cells, while HER2 and MAPK expression levels were substantially increased (Figure 2a,b). This observation suggests that although cells acquire resistance to letrozole while remaining hormone-dependent, they may continue to utilize growth factor signaling pathways for survival. 

### 2.2. Estrogen-Dependent Letrozole-Resistant Cells Are Non-Migratory

**Figure A1:**
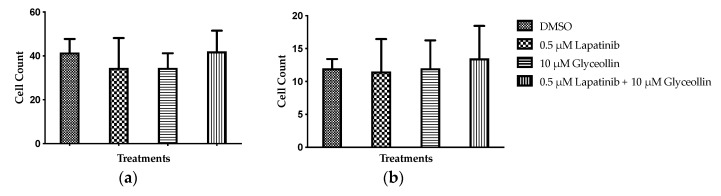
Glyceollin and/or lapatinib does not alter T47Darom or T47DaromLR cell migration. Migration assays were performed using (**a**) the T47Darom cells and (**b**) the T47DaromLR breast cancer cells. Both cell lines were cultured in standard growth media and treated for 24 h with DMSO vehicle control, 0.5 μM lapatinib, 10 μM glyceollin, or 0.5 μM lapatinib 10 μM glyceollin as described in the Section 4. Cells were stained using 0.5% crystal violet in 1% methanol, and graphs depict the number of cells that migrated after 24 h. Student’s *t*-tests were conducted, and treatments were compared to the control. Results are expressed as the mean unit ± SD of three independent experiments in triplicate.

Since the T47DaromLR cells exhibited increased MAPK and HER2 expression, we were interested in determining whether this translated to increased motility, as this was previously observed with the estrogen-independent letrozole-resistant breast cancer cells [5]. Therefore, we performed Boyden chamber motility assays to compare the migration of the T47Darom cells to the T47DaromLR cells. However, upon examination, the T47DaromLR cells were non-migratory, and neither aromatase overexpression nor increased MAPK and HER2 altered motility (Figure A1). While unexpected, it was not surprising since the T47DaromLR cell line is a derivative of the non-migratory T47D parental cells. This indicates that alterations in signaling cascades associated with kinases or growth factor signaling and enhanced motility are mutually exclusive. Additionally, these hormone-dependent, non-migratory cells may represent an early stage of letrozole resistance prior to cells transitioning to a more aggressive and metastatic phenotype.

### 2.3. Glyceollin and Lapatinib Treatment Inhibits Proliferation of Letrozole-Resistant Breast Cancer Cells

**Figure 3:**
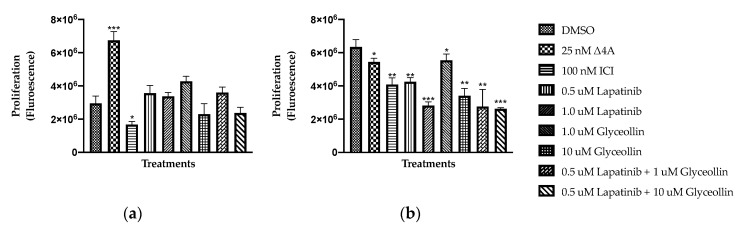
Letrozole-resistant cells are more sensitive to the growth inhibitory effects of glyceollin and/or lapatinib. (**a**) The T47Darom cells were cultured in standard growth media and transferred to phenol red free media for two days prior to treatment with DMSO vehicle control, 25 nM androstenedione, 100 nM ICI, 0.5 μM or 1.0 μM lapatinib, 1 μM or 10 μM glyceollin, 0.5 μM lapatinib ± 1 μM glyceollin or 10 μM glyceollin, and cell proliferation assays were performed after 5 days. (**b**) The T47DaromLR were withdrawn from letrozole for 7 days prior to treatment and treated as indicated above. Proliferation was measured using the resazurin assay, and graphs depict the fluorescence intensity of cells read at 430/560 nM wavelengths over the course of 5 days. Student’s *t*-tests were performed, and treatments were compared to the DMSO control. Results are expressed as the mean unit ± SD (*** *p* < 0.001, ** *p* < 0.01, * *p* < 0.05) of three independent experiments in triplicate.

Our previous studies showed that 10 μM glyceollin inhibits the proliferation of hormone-independent letrozole-resistant LTLT-Ca cells [7]; however, the efficacy of glyceollin on estrogen-dependent letrozole resistance remains unclear. As hormone-dependent T47DaromLR cells express higher levels of MAPK and HER2 compared to the T47Darom cells, we chose to measure the impact of cell proliferation in response to glyceollin and/or lapatinib (a dual EGFR and HER2 inhibitor) treatment. Since both cell lines were ER+ and expressed aromatase, they were treated with the aromatase substrate, androstenedione, to examine aromatase activity and measure estrogen-dependent proliferation. As shown in Figure 3a, androstenedione treatment caused a 129% increase in the proliferation of the T47Darom cells. This indicated that the aromatase protein was functional as it converted androstenedione to estrogen and enhanced proliferation. Interestingly, androstenedione treatment resulted in a 14% decrease in the proliferation of the T47DaromLR cells, which corresponds to decreased aromatase activity and expression in these cells (Figure 3b). 

To evaluate the impact of ER degradation on cell proliferation, both cell lines were treated with fulvestrant (ICI). There was a 44% and 36% decrease in the proliferation of the T47Darom and the T47DaromLR cell lines, respectively, indicating that both cell lines were estrogen-sensitive regardless of letrozole sensitivity and aromatase expression. The growth of the T47Darom cells was insensitive to glyceollin and/or lapatinib treatment, which is likely because the cells were not challenged with estradiol or androstenedione supplementation. When the T47DaromLR cells were treated with both the low and high doses of lapatinib, there was a dose-dependent decrease in cell proliferation. For the remainder of the studies, we chose to use the 0.5 μM dose of lapatinib because it was the lowest dose that inhibited proliferation. This was critical as we planned to combine lapatinib with glyceollin and avoid dose-dependent toxicity when cells were treated with multiple agents. As a single agent, glyceollin had no effect on the T47Darom cells but induced a dose-dependent decrease ranging from 13 to 46% on the proliferation of the T47DaromLR cells. We chose to use the 10 μM dose for the remaining experiments based on the results of the proliferation studies along our previous results in other AI-resistant breast cancer cell lines [18]. Using a combination of lapatinib and glyceollin further decreased proliferation by 56–59% in the resistant cells. While the proliferation of both cell lines was estrogen-dependent, as indicated by their response to fulvestrant, the letrozole-resistant cells were more sensitive to the growth inhibitory effects of glyceollin and lapatinib with combination treatment most effective. These findings highlight the potential of the combination treatment to selectively target AI-refractory cells. Further experiments were conducted using a dose of 0.5 µM lapatinib and 10 µM glyceollin.

### 2.4. Glyceollin and Lapatinib Dramatically Inhibit Survival of Letrozole-Resistant Breast Cancer Cells

**Figure 4:**
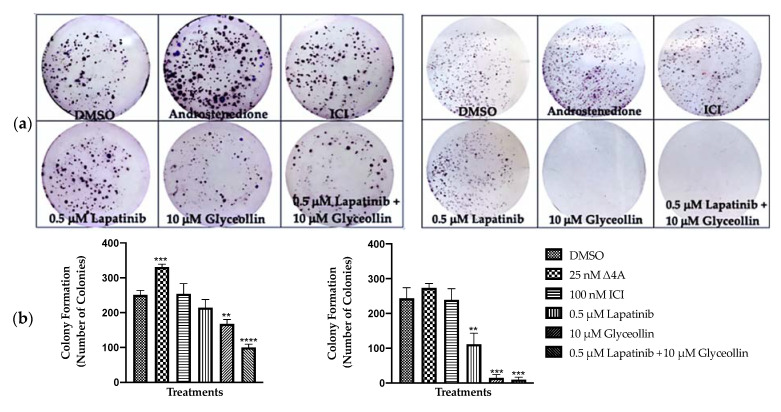
Glyceollin and Lapatinib dramatically inhibit the survival of letrozole-resistant breast cancer cells. Colony formation assays were performed using (**a**) the T47Darom cells and (**b**) the T47DaromLR breast cancer cells. Both cell lines were cultured in standard growth media and treated for 2 weeks with DMSO vehicle control, 25 nM androstenedione, 100 nM ICI, 0.5 μM or 1.0 μM lapatinib, 1 μM or 10 μM glyceollin, 0.5 μM lapatinib ± 1 μM glyceollin or 10 μM glyceollin as described in the Section 4. Cells were stained using 0.5% crystal violet in 1% methanol, and graphs depict the number of cells that formed colonies after 2 weeks. Student’s *t*-tests were performed, and treatments were compared to the DMSO control. Results are expressed as the mean unit ± SD (**** *p* < 0.0001, *** *p* < 0.001, ** *p* < 0.01) of three independent experiments in triplicate.

Our previous report demonstrated that glyceollins inhibit the colony formation of estrogen-independent, letrozole-resistant breast cancer cells [7]; however, its effects on estrogen-dependent, letrozole-resistant breast cancer cells are unclear. We chose to perform colony formation assays in addition to proliferation assays, as the latter relies on metabolically active cells including those that are senescent. Since prematurely senescent cells do not form colonies, colony formation assays were conducted to study the long-term effects of glyceollin and/or lapatinib on the survival of the T47Darom and the T47DaromLR cells. As shown in Figure 4, androstenedione demonstrated a 32% increase in the colony formation of both the T47Darom and T47DaromLR cells, while there was no significant change with fulvestrant treatment in either cell line. As expected, lapatinib alone did not cause a significant change in the colony formation of T47Darom cells as they do not overexpress EGFR or HER2; however, it caused a 54% decrease in the T47DaromLR cell survival. Glyceollin alone decreased survival by 33% and 94% in the T47Darom and the T47DaromLR cell lines, respectively, while the combination treatment caused a 60% and 96% reduction in both the T47Darom and the T47DaromLR cells, respectively. Overall, glyceollin alone and in combination with lapatinib induced cytostatic effects in the T47Darom cells and cytotoxic effects in T47DaromLR cells. 

**Figure A2:**
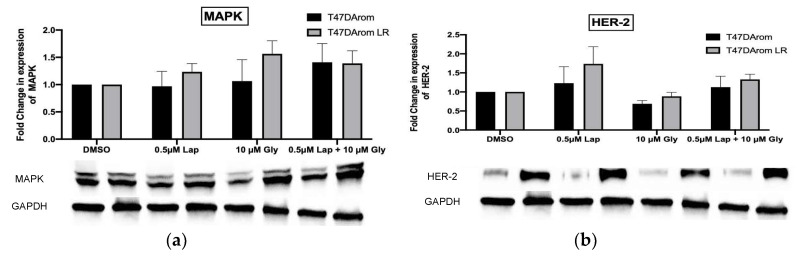
Glyceollin ± lapatinib does not alter the expression of MAPK or HER-2. Both cell lines were cultured in standard growth media and treated for 24 h with DMSO vehicle control, 0.5 μM lapatinib, 10 μM glyceollin, or 0.5 μM lapatinib 10 μM glyceollin as described in the Section 4. Western blot analyses were performed to examine the expression of (**a**) MAPK and (**b**) HER-2. Student’s *t*-tests were performed, and treatments were compared to the control. Densitometry was conducted as previously described. Representative images of the blots are shown below the graphed values.

Since acquisition of resistance is associated with increased MAPK and HER2 (Figure 2) and activation of p38/MAPK signaling cascade [15], we were interested in examining if the glyceollin + lapatinib induced growth suppression was due to decreased activation of MAPK and/or HER. When the cells were treated with glyceollin and/or lapatinib, there was not a significant change in HER2 and MAPK activation (Figure A2), suggesting this may represent one mechanism of resistance but may not indicate the causative inhibitory mechanism by glyceollin even though our previous report demonstrates that the T47DaromLR cells have higher levels of activated phospho-p38 compared to the T47Darom cells [15]. 

### 2.5. Glyceollin and Lapatinib Induce Cell Cycle Arrest in Letrozole-Resistant Breast Cancer Cells

**Figure 5:**
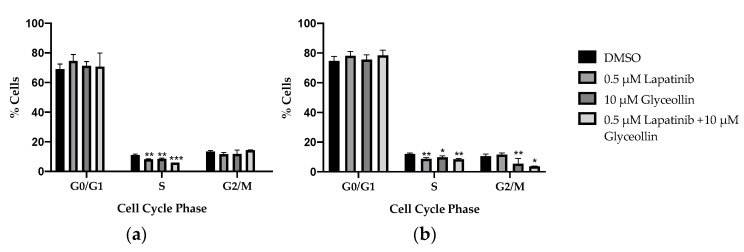
Glyceollin alone and in combination with lapatinib inhibit G1/S and G2/M cell cycle progression in T47DaromLR breast cancer cells. (**a**) The T47Darom and (**b**) the T47DaromLR cells were assessed by flow cytometry for cell cycle progression after treatment with the vehicle control (DMSO), 0.5 μM lapatinib, 10 μM glyceollin, and 0.5 μM lapatinib + 10 μM glyceollin for 24 h. Cells were detached, washed, fixed, and stained with PI. Cell cycle analysis was determined as described in the methodology. Student’s *t*-tests were performed, and treatments were compared to the DMSO control. Results are expressed as a percentage of total cells ± SD (*** *p* < 0.001, ** *p* < 0.01, * *p* < 0.05) of three independent experiments in triplicate.

Previous reports show that AI resistance is due, in part, to cell cycle dysregulation caused by upregulation of cytoplasmic cyclin E [19]. Additionally, glyceollin has been shown to inhibit G1/S phase cell cycle progression in human LNCaP prostate cancer cells [9] and long-term estrogen-deprived (LTED) MCF-7 breast cancer cells [20]. In order to determine if the glyceollin-induced decrease in cell viability is a consequence of cell cycle dysregulation, flow cytometric analyses were performed to ascertain the effects of combination therapy on the cell cycle phase distribution of the letrozole-sensitive and letrozole-resistant breast cancer cells (Table 1). Regardless of the sensitivity to letrozole, results demonstrate that while there was a significant decrease in the percentage of all cells transitioning from G1 to S phase, lapatinib ± glyceollin significantly retarded entry into the S phase (Figure 5). Compared to the vehicle control, glyceollin ± lapatinib had no effect on the T47Darom cells once they entered the G2/M phase. Interestingly, glyceollin and glyceollin ± lapatinib treatment inhibited the entry of the T47Darom cells from entering the G2/M phase. It is possible that the drug-induced cell cycle arrest contributes to the reduction in proliferation and survival, thereby implicating this as a potential inhibitory mechanism.

### 2.6. Glyceollin Induces Apoptosis in Letrozole-Sensitive and Letrozole-Resistant Breast Cancer Cells

**Figure 6:**
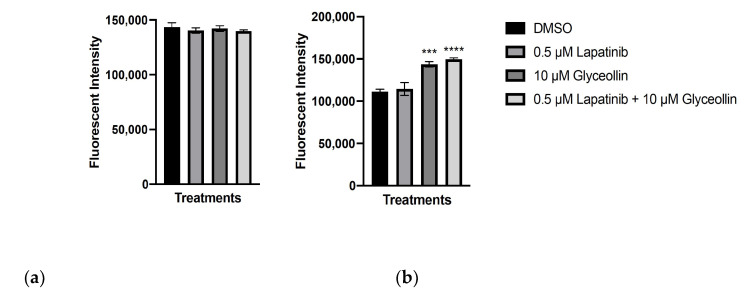
Glyceollin induces apoptosis regardless of aromatase inhibitor sensitivity. (**a**) The T47Darom and (**b**) T47DaromLR cells were used to measure caspase 3/7 activity. T47DaromLR cells were withdrawn from letrozole for 7 days prior to treatment. Cells were treated with control (DMSO), 0.5 μM lapatinib, 10 μM glyceollin, and 0.5 μM lapatinib in combination with 10 μM glyceollin, and apoptosis studies were performed. Caspase activity was measured by reading the fluorescence of cells read at 530/502 nM wavelengths. Student’s *t*-tests were performed, and treatments were compared to the DMSO control. Results are expressed as the mean unit ± SD (**** *p* < 0.0001, *** *p* < 0.001) of three independent experiments in triplicate.

Measurement of apoptosis was performed to ascertain the underlying mechanism responsible for the reduction in the proliferation, viability, and cell cycle arrest of the breast cancer cells following glyceollin and lapatinib treatment. Apoptosis was measured using the Caspase 3/7 assay where the T47Darom and T47DaromLR cells were treated with glyceollin ± lapatinib for 5 days and exposed to the caspase 3/7 detection reagent. This non-fluorescent reagent contains a four amino acid peptide consisting of a cleavage site for caspase 3/7 that inhibits the ability of the dye to bind to DNA. Once caspase 3/7 is detected, the peptide is cleaved which allows the dye to bind DNA and produce a fluorogenic response. As shown in Figure 6a, drug treatment did not affect the T47Darom cells. Interestingly, when the T47DaromLR cells were treated with glyceollin alone or in combination with lapatinib, there was a modest induction of apoptosis by 1.29-fold and 1.35-fold, respectively (Figure 6b). The T47DaromLR cells responded similarly when treated with glyceollin alone and the combination of glyceollin and lapatinib, indicating this effect is likely due to glyceollin. Lapatinib alone had no effect on apoptosis.

**Figure 7:**
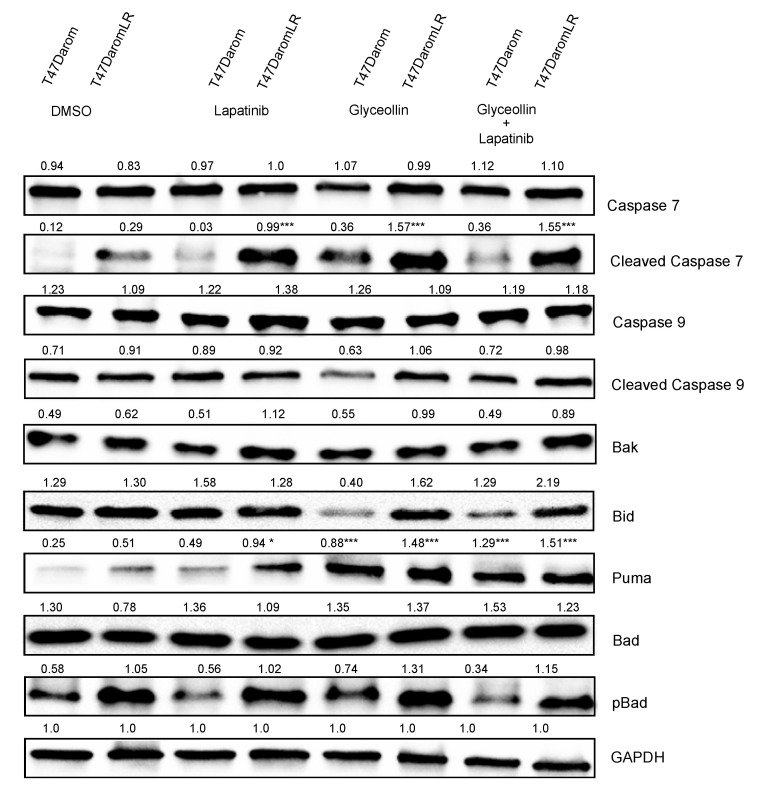
Western blot analysis for apoptosis-related proteins. T47Darom and T47DaromLR cells were exposed to DMSO, 0.5 μM lapatinib, 10 μM glyceollin, and 0.5 μM lapatinib in combination with 10 μM glyceollin for 24 h, and GAPDH was used as the loading control. Densitometry values are indicated above each band, and the treatments were compared to DMSO control for the respective cell lines and normalized to GAPDH loading control. Student’s *t*-tests were performed, and results are expressed as the mean (*** *p* < 0.001, * *p* < 0.05) of a minimum of three independent experiments in triplicate.

Since glyceollin modestly induced changes in caspase 3/7 activity, we sought to identify which event(s) in the glyceollin-treated cells led to caspase activation and apoptotic cell death. Therefore, immunoblots were performed to examine the expression of various caspases and proteins involved in apoptosis in the presence or absence of treatment. Initially, we examined the expression of BH3 only pro-apoptotic family members (Bid, Puma, and Bad). Puma was measured and endogenous levels were higher in the T47DaromLR cells. When both cell lines were treated with glyceollin ± lapatinib, Puma expression was further increased. Bid was also measured as it is a sensor that inactivates anti-apoptotic proteins and activates pro-apoptotic proteins. Glyceollin and glyceollin + lapatinib treatment led to decreased Bid expression in the T47Darom cells while Bid expression persisted in the T47DaromLR cells in the presence of drug treatment. Bad expression was also examined, and although total levels of Bad were similar between both cell lines, compared to the T47Darom cells, the T47DaromLR cells expressed higher endogenous levels of pBad regardless of treatments. We then measured the expression of Bak, a downstream pro-apoptotic effector, and the T47DaromLR cells expressed higher levels of Bak in the presence or absence of treatment. To confirm the caspase 3/7 assay results, both cell lines were treated with glyceollin ± lapatinib and total levels of caspase 7 were not significantly different. Interestingly, when both cell lines were compared, the T47DaromLR cells expressed higher endogenous levels of cleaved caspase 7 (Figure 7). In the presence of glyceollin ± lapatinib, cleaved caspase 7 was significantly increased in the T47DaromLR cells while drug treatment had no effect on cleaved caspase 7 in the T47Darom cells. The initiator caspase (i.e., caspase 9) was also examined, but there were no significant changes in the expression of the full-length or cleaved forms. Taken together, activation of caspase 7 in the glyceollin-treated T47DaromLR cells is a result of activation of the intrinsic apoptotic signaling cascade.

## 3. Discussion

Previous reports from our lab demonstrated that glyceollin alone inhibited proliferation and reversed the epithelial-to-mesenchymal transition in hormone-independent letrozole-resistant (LTLT-Ca) breast cancer cells [7]. However, less is known about the impact of glyceollin in the hormone-dependent AI-refractory setting. Although glyceollins alone have shown promise in the endocrine-resistant preclinical setting [8], it is unlikely that a single phytochemical would significantly inhibit growth and metastasis in the clinical setting. As such, this study was designed to provide mechanistic insight into the inhibitory effects of glyceollin alone and in combination with lapatinib in estrogen-dependent AI-resistant breast cancer cells. For these studies, we utilized a letrozole-sensitive (T47Darom) and letrozole-refractory (T47DaromLR) cell line derived from the T47D ductal carcinoma cell line. While this study is limited by the use of one AI-sensitive and -resistant cell model, previous studies by our group examined other letrozole-resistant cell lines with varying molecular characteristics such as loss of the ER and hormone independence [5,7,21]; this cell line represents a unique phenotype compared to previously examined cell lines. Upon characterizing the cells, it was interesting to find that like the previously tested letrozole-resistant LTLT-Ca breast cancer cells, the T47DaromLR cells overexpressed MAPK and HER2. Unlike the LTLT-Ca cells, the T47DaromLR cells retained the ER and remain sensitive to the growth inhibitory effects of fulvestrant. In our previously published study, using the T47D-derived cell lines, immunofluorescence staining was performed to compare ER localization [22]. When compared to the T47Dcon cells, both the T47Darom and T47DaromLR cells, which express increased aromatase, exhibited increased nuclear localization of the ER in the absence of hormone treatment. This suggests that while the T47DaromLR cells are ER+, sensitive to the anti-proliferative effects of fulvestrant, and exhibit ER translocation in the absence of hormone, this cell line represents an intermediate AI-resistant phenotype that has a heterogeneous population of cells that are partially hormone-dependent and -independent.

As resazurin assays were used to measure proliferation, colony formation assays were used to examine the long-term cell survival after therapy. Interestingly, in some instances, each approach caused a different effect. Androstenedione treatment caused a slight inhibition in proliferation in the T47DaromLR cells while it had no effect on colony formation. Our previously published findings demonstrate that androgens can induce or inhibit the growth of breast cancer cells, that is, depending upon the estrogen milieu [23]. It was reported that any change in the balance between estrogenic and androgenic influences alters growth. Here, androstenedione treatment shifted the balance to an inhibitory state as a result of low estrogen milieu mediated by long-term letrozole exposure. Fulvestrant treatment inhibited the proliferation of the T47DaromLR cells but did not alter colony formation, thus indicating the potential of the T47DaromLR cells to develop cross-resistance to fulvestrant in the long-term setting. The growth inhibitory properties of the combination of lapatinib and glyceollin treatment of the T47DaromLR cells were not time-dependent, suggesting that these cells are less likely to develop cross resistance to this therapy after long-term treatment. 

While glyceollins have shown promise in the in vitro and in vivo settings, due to the poor bioavailability of most phytochemicals, there are concerns as to whether glyceollins can reach the mammary tissue. Unfortunately, there are limited human studies documenting dietary phenolic compounds and mammary gland distribution. A recent review article describes many of the dietary phenolic compounds and derived metabolites identified in human breast tissues, but many of these human intervention studies were limited by small sample sizes [24]. However, several primate studies were conducted to overcome these limitations and evaluate the bioavailability of glyceollins given with a soy protein matrix [25,26,27]. Thirty female, postmenopausal cynomolgus macaques were randomized and treated with control (1mg/day estradiol + casein/lactalbumin), 1 mg/day estradiol + soy protein isolate (SPI) containing 194 mg/day isoflavonoids, and 1 mg/day estradiol + glyceollin-enriched soy protein containing 189 mg/day isoflavones +134 mg/day glyceollins. The glyceollin dose that was used in the study is theoretically attainable via dietary means. The dietary glyceollins from elicited soy protein were absorbed and present at serum concentrations at 4 h post-feeding and negligible concentrations in the SPI group. When given with estradiol, glyceollin-enriched protein resulted in a modest reduction of estrogenic stimulation in breast as well as a reduction in mammary gland expression of two estrogen-responsive genes (i.e., trefoil factor 1 and progesterone receptor), suggesting that glyceollins competitively bind to the estrogen receptor in the breast. While these preliminary reports and reviews suggest that dietary glyceollins target the mammary gland and may prove useful when combined with targeted therapy [28], clinical use remains hampered by limited investigation in human trials.

Previous reports demonstrate that glyceollins inhibit estrogen-dependent growth of MCF-7 cells and T47D cells [18,29]. However, in the absence of estrogen stimulation, glyceollin had no effect on the growth and viability of the T47Darom cells suggesting that in these cells, glyceollin is a competitive inhibitor of estradiol. Interestingly, this was not the case when the T47DaromLR cells were treated with glyceollins. In the absence of estrogen stimulation, glyceollins reduced proliferation and colony formation in the T47DaromLR cells suggesting an estrogen-independent growth inhibitory mechanism. 

The T47DaromLR cells are ER-positive, yet, have increased growth factor expression which suggests a partial dependence on both estrogen-mediated proliferation and growth factor signaling pathways for survival; however, these ductal cells are non-migratory. Considering both factors implies that the T47DaromLR cells may represent an early stage of letrozole resistance. Since we previously demonstrated that glyceollins reversed EMT in the letrozole-resistant LTLT-Ca cells, we were interested in determining if there were additional mechanisms unrelated to EMT by which glyceollin and lapatinib could exert inhibitory effects using a non-migratory letrozole-resistant breast cancer cell line. Although many of the cells were present in the G1 phase, glyceollin and the combination therapy prevented the T47DaromLR cells from entering the G2/M phase. The ability of glyceollin and the combination therapy to prevent both S and G2/M phase entry may account, in part, for the dramatic reduction in the viability of the T47DaromLR cells compared to the T47Darom cells. Additionally, glyceollin treatment selectively induced apoptosis in the T47DaromLR cells but not the T47Darom cells. Lapatinib alone had no effect on apoptosis in either cell line; thus the apoptotic effect observed with combination therapy is likely due to glyceollin alone. The ability of glyceollin and lapatinib treatment to both inhibit apoptosis and prevent cells from entering the S and G2/M may account for the reduced proliferation and cytotoxic effects observed in the T47DaromLR cells. While other studies highlight the antiestrogenic effects of glyceollin, here we showed that in the absence of estrogen, glyceollin and lapatinib have the potential to inhibit proliferation and viability, disrupt the cell cycle, and induce apoptosis.

**Figure 8:**
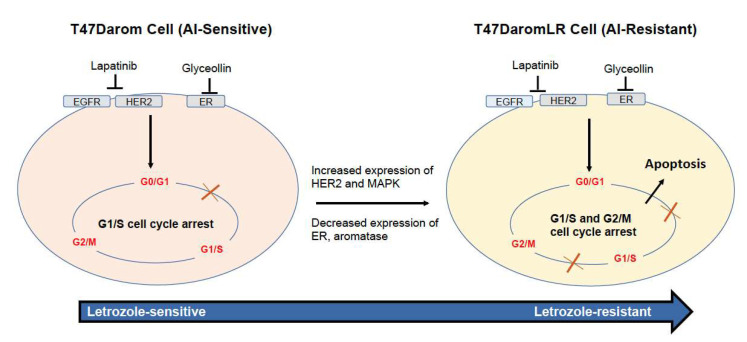
Schematic representing the glyceollin-induced inhibitory pathway in the T47DaromLR cell lines. Glyceollin blocks the ER while lapatinib blocks the EGFR and HER2 receptors leading to activation of caspase 3/7 activity, activation of pro-apoptotic proteins, and inhibition of anti-apoptotic proteins which leads to decreased proliferation and survival.

Since glyceollin induced caspase 3/7 activation, we chose to examine what upstream events were responsible for this effect. As cells acquire resistance to letrozole, they begin to adapt to their new milieu by increasing growth factoring signaling which is inherently associated with cellular stress. In response to the acquired-resistance-induced stress, pro-apoptotic BH3 family members (Bim, Bid, Puma, pBad) were upregulated and activated causing activation of the pro-apoptotic effector Bak. Since activation of Bak mediates the release of cytochrome C and ultimately activates caspase signaling, it was not surprising that cleaved caspase 7 was induced in the letrozole-resistant cells and led to cell cycle arrest and cellular death. As both hormone-dependent and hormone-independent letrozole resistant breast cells respond to the inhibitory effects of glyceollins, this is the first study that distinguishes how glyceollins exert their effects on hormone-sensitive AI-resistant breast cancer cell lines (Figure 8).

## 4. Materials and Methods 

### 4.1. Cell Culture

In this study, we utilized two cell lines previously developed and derived from the T47D parental breast cancer cell line [30]. The T47Darom (T47D cells stably transfected with the human aromatase gene) were cultured and maintained in phenol red DMEM media (Life Technologies, Carlsbad, CA, USA) supplemented with 5% fetal bovine serum (FBS), (VWR, Radnor, PA, USA), penicillin-streptomycin, antimycotic-antibiotic (10,000 U/mL penicillin G sodium; 10,000 μg/mL streptomycin sulfate; and 7.5 μg/mL geneticin (Life Technologies, Carlsbad, CA, USA). T47DaromLR (generated by incubating T47Darom for 75 weeks in the presence of 10 μM letrozole) were maintained in phenol red free DMEM media (Life Technologies, Carlsbad, CA, USA) supplemented with 10% charcoal-stripped fetal bovine serum (FBS), (VWR, Radnor, PA, USA), penicillin-streptomycin, antimycotic-antibiotic (10,000 U/mL penicillin G sodium; 10,000 μg/mL streptomycin sulfate; 7.5 μg/mL geneticin (Life Technologies, Carlsbad, CA, USA); and 1 μM letrozole (Sigma-Aldrich, St. Louis, MO, USA). The cells were maintained in a tissue culture incubator in a humidified atmosphere of 5% CO_2_ and 95% air at 37°C. Both cell lines were a generous gift from ITT Research Institute. The T47Darom and T47DaromLR cell lines were authenticated by short tandem repeat (STR), profiling by the American Type Culture Collection (ATCC, Manassas, VA, USA), confirming that they share more than 85% homology with the T47D cell line.

### 4.2. Viability Assay

Proliferation assays were performed as previously described [31]. Specifically, the T47Darom (AI-sensitive) cells were plated in 96-well plates at a density of 2 × 10^3^ cells per well for each cell line and allowed to recover for 24 h. The T47DaromLR (AI-resistant) cells were cultured in the presence of letrozole. For proliferation assays, the cells were withdrawn from letrozole for 168 h and afterwards treated with control (DMSO), 25 nM androstenedione, 100 nM ICI, 0.5 μM lapatinib, 1.0 μM lapatinib, 1.0 μM glyceollin,10 μM glyceollin, 0.5 μM lapatinib plus 1.0 μM glyceollin, or 0.5 μM lapatinib plus 10 μM glyceollin in 96-well plates to determine the effects of the various treatments in the absence of letrozole. The resazurin dye (Sigma-Aldrich, St. Louis, MO, USA) was added to each well at 10% of the total volume and measured at 24, 48, 72, and 120 h. The SpectraMax^®^ MiniMax^®^ 300 Imaging Cytometer (Molecular Devices, San Jose, CA, USA) was used to measure fluorescence and background wavelengths at 530 nm and 590 nm to determine proliferation. All experiments were performed with *n* ≥ 3, and a total of 3 biological replicates were performed. The proliferative activity was calculated as a percent of the vehicle controls as follows:

Antiproliferative activity = [Fluorescence of viable cells (control) − Fluorescence of viable cells (treated)]/Fluorescence of viable cells (control)

### 4.3. Colony Formation Assay

Cells were cultured in standard growth media as described above, and the T47Darom cells were seeded at a density of 1.5 × 10^3^ cells per well in 6-well plates. T47DaromLR cells were seeded at a density of 1.5 × 10^3^ cells per well in 6-well plates after 168 h inhibitor withdrawal treatment. The cells were allowed to attach overnight and treated on the following day with DMSO vehicle, 25 nM androstenedione, 100 nM ICI, 0.5 μM lapatinib, 10 μM glyceollin, or 0.5 μM lapatinib plus 10 μM glyceollin. Media was replaced every 3 days and treated with appropriate drug for 2 weeks. Cells were routinely monitored, and after 2 weeks, the media was removed, and the cells were fixed with formaldehyde and dried overnight. The cells were then washed and stained with crystal violet and dried. Afterwards the colonies were counted using a manual counter and microscopy. Colonies with ≥50 cells were determined to be positive. All experiments were performed with *n* ≥ 6, and a total of 3 biological replicates were performed.

### 4.4. Migration Assay

For migration assays, the T47Darom and the T47DaromLR cells were plated in 25 cm^2^ flasks and treated with either control (DMSO), 0.5 μM lapatinib, 10 μM glyceollin, or 0.5 μM lapatinib plus 10 μM glyceollin for 24 h. The following day, cells were trypsinized, centrifuged, and resuspended in serum-free media. Afterwards 250 μL of 2.5 × 10^4^ cells was added to the inner chamber of the insert, and 500 μL of either serum-free or serum-containing media was added to the outer chamber. Cells were incubated for 24 h at 37 °C with 5% CO_2_. The media was aspirated, and the cells in the bottom of the well were fixed with 4% formaldehyde for 10 min at room temperature. The formaldehyde was aspirated and stained with crystal violet in 0.5% methanol. Media was aspirated, and cells were re-suspended in serum-free media to the same cell concentration. The upper membrane was scraped to remove non-migratory cells. The membrane was fixed between a glass microscope cover slip using Permount (Fisher Scientific, Hampton, NH, USA). All experiments were performed with *n* ≥ 6, and a total of 3 biological replicates were performed. The images were captured by microscopy using Olympus BX41 (Olympus, Shinjuku-ku, Tokyo, Japan) at 10x magnification. Cells were counted using ImageJ software.

### 4.5. Flow Cytometry

The T47Darom and T47DaromLR cells were seeded at a density of 1.5 × 10^6^ cells in a 75 cm^2^ flask with appropriate media overnight. Cells were synchronized in 1% FBS for 24 h. On the following day, cells were treated with DMSO control, 0.5 μM lapatinib, 10 μM glyceollin, or 0.5 μM lapatinib plus 10 μM glyceollin. Cells were incubated for 24 h at 37 °C with 5% CO_2_. On the following day, the cells were trypsinized, centrifuged at 2500 rpm for 2 min. The media was removed, and cells were resuspended in 5 mL 1X PBS without Mg^2+^ and Ca^2^^+^. Cells were vortexed and centrifuged for 2500 rpm for 2 min. PBS was removed without disturbing the pellet. Approximately 200 to 500 μL of PBS was left in the tube to properly resuspend cells. Cells were vortexed at 3000 rpm and fixed by slowly adding 5 mL of 100% absolute ice-cold ethanol dropwise to the cells. Samples were vortexed and centrifuged at 3000 rpm for 5 min. The ethanol was removed leaving approximately 200 μL to resuspend cells. Cells were briefly vortexed, and 1 mL of Propidium Iodide (PI) solution (100 μL PI stock, 25 mg RNAse A, 5–10 mg glucose or dextrose, and 10 mL 1X PBS (without Mg^2+^ and Ca^2+^)) was added to each sample and vortexed at a speed of 2000 rpm for 1 min. Samples were incubated for 1 h at room temperature. The distribution of DNA in all cell cycle phases was assayed in replicates within 2 h by flow cytometer (FACS caliber, BD Biosciences, San Jose, CA, USA). In each sample, a total of 10,000 individual events were acquired separately, and doublets were removed by gating. BD CellQuest Pro software (BD Biosciences, San Jose, CA, USA) was used for acquisition of the data, and the percentage of cells in each phase was determined using ModFit LT 3.2.1 Software (Verity software house Inc., Topsham, ME, USA). 

### 4.6. Caspase 3/7 Apoptosis Assay

The T47Darom and T47DaromLR cells were seeded at a density of 1.5 × 10^3^ cells in a 96-well plate with appropriate media overnight. Cells were treated the following day with DMSO control, 0.5 μM lapatinib, 10 μM glyceollin, or 0.5 μM lapatinib plus 10 μM glyceollin for 5 days. Following the drug treatment, the cells were treated with 4 μM CellEventTM Caspase-3/7 Green Detection Reagent (Invitrogen, Carlsbad, CA, USA) and incubated for 30 min at 37 °C with 5% CO_2_. The cells were then fixed with 4% formaldehyde and allowed to incubate for 15 min at room temperature. The SpectraMax^®^ MiniMax^®^ 300 Imaging Cytometer (Molecular Devices, San Jose, CA, USA) was used to measure fluorescence and background wavelengths at 530 nm and 502 nm, respectively. Cells were imaged on Nikon Ti Eclipse microscope (Nikon, Minato, Tokyo, Japan) using an FITC filter. 

### 4.7. Gel Electrophoresis and Western Blot Analysis

Briefly, the cells were treated with DMSO control, 0.5 μM lapatinib, 10 μM glyceollin, or 0.5 μM lapatinib plus 10 μM glyceollin for 24 h. Afterwards the cells were gently scraped and homogenized in cold RIPA buffer supplemented with 2× protease and phosphatase inhibitors (Thermo Fisher Scientific, Inc., Waltham, MA, USA). Samples were incubated on ice for 30 min and centrifuged for 20 min at 12,000 rpm. The protein extract was quantified using the Bradford assay. For gel electrophoresis, all samples were incubated with Laemmli protein sample buffer (Bio-Rad, Hercules, CA, USA) at 70 °C for 10 min. Then, 75 µg of denatured protein was separated using 4–20% Mini-PROTEAN^®^ TGX™ Precast Protein Gels (Bio-Rad) and transferred to polyvinylidene difluoride (PVDF) membranes. All blots were blocked for 1 h with 5% bovine serum albumin (BSA) in phosphate-buffered saline (PBS) and 0.1% Tween-20 (PBS-T) buffer. Following incubation with primary antibodies from Cell Signaling Technology (Cell Signaling Technology, Danvers, MA, USA) recognizing ERα (catalog No.: 8644S), HER2 (catalog No.: 2165), MAPK (catalog No.: 4695), EGFR (catalog No.: 4267), Bad (catalog No.: 9239), phospho-Bad (catalog No.: 5284), Bak (catalog No.: 12105), Bid (catalog No.: 2002), Puma (catalog No.: 12450) caspase 7 (catalog No.: 12827), cleaved caspase 7 (catalog No.: 8438), caspase 9 (catalog No.: 9508), cleaved caspase 9 (catalog No.: 52873), and GAPDH (catalog No.: 2118). The following antibody was purchased from Abcam: aromatase (catalog No.: ab18995). The membranes were incubated with the anti-rabbit secondary antibody (catalog No.: 7074S). The protein bands were detected using the Clarity Max Western ECL Substrate (Bio-Rad) according to the manufacturer’s instructions. Immunoreactive bands were visualized using the ChemiDoc XRS imaging system (Bio-Rad). The exposure time was automatically detected by the imaging system. The protein bands were analyzed using Image Lab software (Bio-Rad). Arbitrary densitometry units were quantified and expressed as mean ± standard deviation. The bands were normalized to the housekeeping protein bands (GAPDH), whereby the density of the target protein in each lane was multiplied by the ratio of the loading control density from the control sample to the loading control density of other lanes. The immunoblot images are representative of more than three independent experiments with a minimum of 2 duplicates per sample.

### 4.8. Statistical Analysis

All data are presented as mean ± SD using the Graph Pad Prism V.8 software program from minimum of three independent observations. Statistical differences between the samples were determined by Student’s *t*-test where *p* < 0.05 was considered as significant. Results are expressed as the mean unit ± SD (*** *p* < 0.001, ** *p* < 0.01, * *p* < 0.05) where treatments were compared to the DMSO control.

## Figures and Tables

**Table 1:** Glyceollin and lapatinib alter cell cycle distribution. This table illustrates the number of cells analyzed and percent distribution of cells entering the various stages of the cell cycle. Numbers in parentheses indicate the total number of cells in each phase.

Cell Line	G0/G1	S	G2/M	Total Number of Cells
T47Darom				
Control	70.00% (8413)	11.60% (1392)	12.90% (1553)	11358
Lapatinib	74.80% (7231)	8.10% (783)	12.50% (1212)	9226
Glyceollin	72.30% (6363)	8.10% (709)	13.70% (1210)	8282
Lapatinib + Glyceollin	66.20% (5850)	6.80% (604)	14.30% (1266)	7720
T47DaromLR				
Control	75.55% (5857)	10.05% (779)	13.85% (1074)	7753
Lapatinib	79.85% (5828)	7.65% (560)	11.60% (850)	7323
Glyceollin	75.85% (6084)	8.8% (706)	7.3% (8585)	8021

## Data Availability

Not applicable.

## Abbreviations

AI	aromatase inhibitors
Ca^2+^	calcium ion
CO_2_	carbon dioxide
DMSO	dimethyl sulfoxide
EGFR	epidermal growth factor receptor
ER-	estrogen receptor negative
ER+	estrogen receptor positive
EMT	epithelial-to-mesenchymal-transition
FBS	fetal bovine serum
FDA	Federal Drug Administration
HER2	human epidermal growth factor receptor
HR+	hormone receptor positive
LTED	long-term estrogen-deprived
LTLT-Ca	long-term letrozole-treated
MAPK	mitogen-activated protein kinase
Mg^2+^	magnesium ion
PBS	phosphate-buffered saline
PI	propidium iodine
PR+	progesterone receptor positive
RPM	revolutions per minute
T47Darom	T47Daromatase-overexpressing cells
T47DaromLR	T47D-aromatase-overexpressing letrozole-resistant cells
